# Takotsubo syndrome triggered by a pleasant emotional event: a case report

**DOI:** 10.47487/apcyccv.v6i3.500

**Published:** 2025-09-24

**Authors:** Eder Jonathan Amaro-Palomo, Myles Andrew Laurence-Salinas, Santiago Alba-Valencia, Diego Araiza-Garaygordobil

**Affiliations:** 1 Coronary Care Unit, Instituto Nacional de Cardiología «Ignacio Chávez», Mexico City, Mexico. Coronary Care Unit Instituto Nacional de Cardiología «Ignacio Chávez Mexico City Mexico

**Keywords:** Takotsubo Syndrome, Apical Ballooning Syndrome, Cardiology, Case Report, Síndrome de Takotsubo, Síndrome de Balonamiento Apical, Cardiología, Reporte de caso

## Abstract

Takotsubo Syndrome (TTS) is characterized by transient left ventricular dysfunction, often triggered by emotional or physical stress. While negative emotional events are common triggers, positive emotional events can also induce a rare variant known as Happy Heart Syndrome (HHS). This case report describes a 53-year-old male who presented with acute chest pain following a positive emotional stimulus: a birthday call offering him an opportunity to play with his favorite music group. Initial workup suggested an acute myocardial infarction, but coronary angiography revealed no obstruction. Left ventriculography showed the classic apical ballooning pattern of TTS. The patient was diagnosed with HHS. This case underscores the importance of considering positive emotional triggers in the diagnosis of TTS and highlights the need for further research into its diverse presentations and management strategies.

## RESUMEN

El síndrome de Takotsubo (TTS) se caracteriza por la disfunción transitoria del ventrículo izquierdo, a menudo desencadenada por estrés emocional o físico. Si bien los eventos emocionales negativos son desencadenantes comunes, los eventos emocionales positivos también pueden inducir una rara variante conocida como el síndrome del corazón feliz (HHS). Este informe de caso describe a un hombre de 53 años que presentó dolor torácico agudo después de un estímulo emocional positivo: una llamada de cumpleaños que le ofrecía la oportunidad de tocar con su grupo musical favorito. El estudio inicial sugirió un infarto agudo de miocardio, pero la angiografía coronaria no mostró obstrucción. La ventriculografía izquierda mostró el clásico patrón de globos apicales de TTS. El paciente fue diagnosticado con HHS. Este caso subraya la importancia de considerar los desencadenantes emocionales positivos en el diagnóstico de TTS y resalta la necesidad de más investigaciones sobre sus diversas presentaciones y estrategias de manejo.

## Introduction

**Figure 1:**
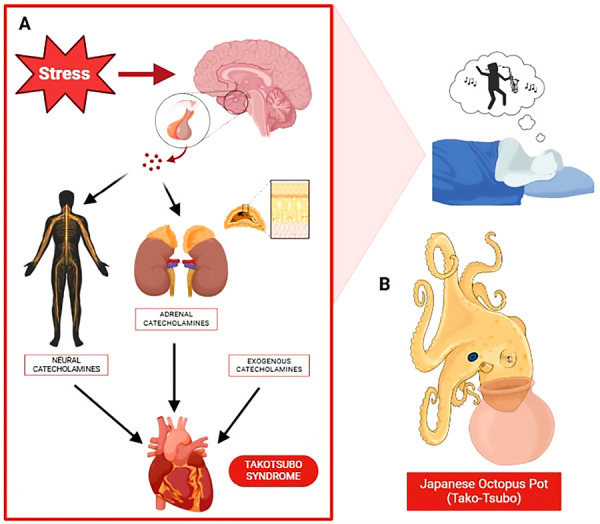
A) TTS is triggered by emotional stress, catecholamine release, resulting in temporary structural dysfunction. B) Japanese octopus pot.

Takotsubo Syndrome (TTS) was first identified in 1990 by Sato *et al.* in Japan, described as sudden, temporary systolic dysfunction of the left ventricle, characterized by a typical pattern of anteroseptal and apical dyskinesia, with hyperkinesia of basal segments, in the absence of obstructive coronary artery disease, which takes on a shape resembling a Japanese octopus trap, known as “Takot-tsubo” identified on ventriculography. ^1^ (Figure 1).

Stress induced from birthdays may trigger vascular events such as stroke or acute myocardial infarction in patients with predisposing conditions. ^1^ Positive emotional events can also induce a rare variant of TTS known as “happy heart syndrome” (HHS), where triggers are reported to include pleasant emotional experiences. This highlights the need to identify distinct patient clusters to tailor management strategies. ^1^^-^^2^

This report will describe what a medical practitioner should consider when encountering a case of acute chest pain, dyspnea, or other symptoms that could mimic a coronary syndrome, especially when triggered by a positive emotional event, as opposed to the more common negative emotional triggers typically associated with TTS. Additionally, it illustrates how an intense positive emotional response can lead to a potentially life-threatening condition, specifically after a birthday surprise, an HHS.

## Case Report

A 53-year-old male musician with a history of dyslipidemia, no smoking, and no cardiovascular history presented to the emergency department after waking up with sudden, intense, oppressive chest pain accompanied by nausea and a single episode of vomiting. Physical examination revealed an elevated heart rate of 110 bpm.

**Figure 2:**
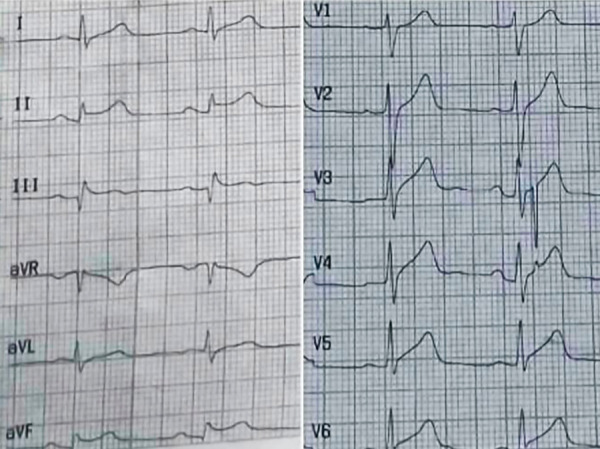
Initial ECG. Q-waves and ST-segment elevation in the inferior leads, with an elevation of 2 mm in DIII.

A 12-lead electrocardiogram (ECG) revealed sinus tachycardia, a normal axis, Q-waves, and ST-segment elevation in the inferior leads, with an elevation of 2 mm in DIII. Additionally, there was an elevation of 0.75 mm in the V7-V9 leads, and a normal QTc interval of 422 ms. (Figure 2)

Laboratory blood workup showed elevated levels of high-sensitivity troponin I (973 ng/L; normal ≤14 ng/L), creatine kinase-MB (CKMB) (81.8 U/L; normal 5-25 U/L), total creatine kinase (885 U/L; normal 10-149 U/L), and N-terminal pro-B-type natriuretic peptide (NT-ProBNP) (1627 pg/L; normal 5-175 pg/mL). Leukocyte count was 8130/µL, lactate was 1.5 mmol/L, and creatinine was 0.9 mg/dL. The remaining laboratory results were within normal parameters.

**Figure 3:**
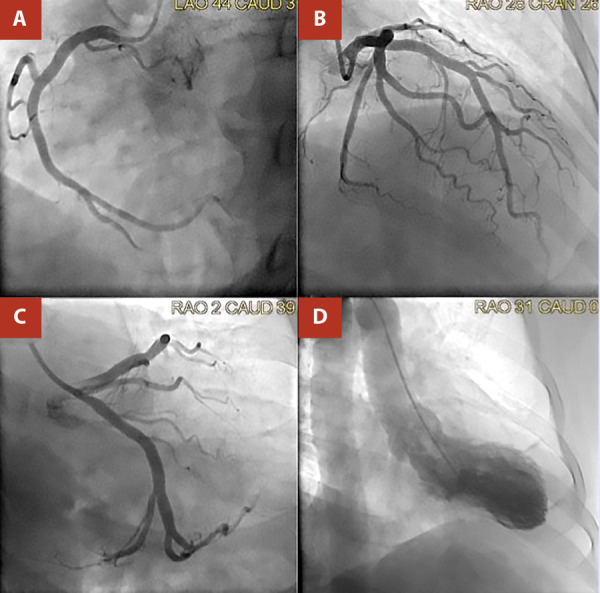
Angiography without evidence of coronary obstruction in A) Right coronary artery, **B)** Anterior interventricular artery. **C)** circumflex artery. **D)** Ventriculography showed ballooning, apical hypokinesis, and basal hypercontractility, a typical pattern of Takotsubo syndrome.

Due to the suspicion of ST-elevated myocardial infarction, the patient was quickly transferred to the cardiac catheterization laboratory, where coronary angiography showed an absence of coronary obstruction. A left ventriculography was performed, demonstrating left ventricular apical ballooning hypokinesia and a hypercontractile base, compatible with a typical pattern of TTS, measuring a 25-mmHg diastolic-to-systolic pressure difference. (Figure 3)

The patient was admitted to the coronary care unit. Management included a beta-blocker (carvedilol 6.25 mg) for rate control and sympathetic modulation. Given the persistently elevated heart rate and the goal of optimizing rate control without compromising contractility, ivabradine was added. Prophylactic anticoagulation with apixaban 5 mg was initiated due to apical hypokinesia and the associated risk of thrombus formation.

A transthoracic echocardiogram (TTE) revealed hypokinesia of the four apical segments of the left ventricle and apex, hypercontractile basal segments with no dynamic intraventricular obstruction. Mild left ventricular systolic dysfunction with an ejection fraction (LVEF) of 50% and a global longitudinal strain of -13.7% was noted. No valvular abnormalities, pericardial effusion, or mural thrombus were visualized.

A history obtained from the patient’s family revealed that the patient had received a birthday call at midnight with the news that, as a birthday gift, he would have the opportunity to play saxophone with his favorite renowned music group. This caused him to wake up in the early hours of the morning with chest pain. According to the criteria and diagnostic algorithm set by the Heart Failure Association Takotsubo Syndrome Taskforce of the European Society of Cardiology, a diagnosis of TTS was made, specifically identifying a rare variant known as HHS.

The patient remained asymptomatic and hemodynamically stable without complications or the need for vasopressor therapy during 5 days of hospitalization. He was discharged without any complications.

At 2-month and 6-month follow-up, the patient continued to be asymptomatic. ECG showed sinus rhythm without abnormalities, laboratory results were within reference parameters, and LVEF was 55%, so it was decided to discontinue anticoagulation.

**Figure 4:**
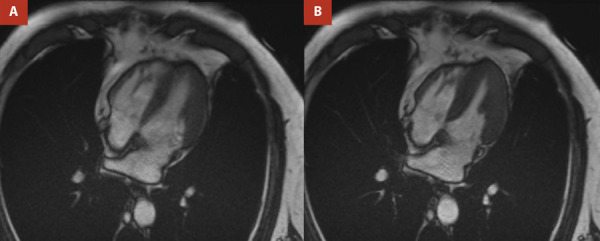
Cardiac MRI. *Panels* **A-B.** Cardiac chamber dimensions are within normal limits. Left ventricle with no dilatation and a preserved LVEF of 54%. No evidence of a linear ischemic pattern in the anterior or septal walls is observed.

Consistent with data showing no difference in in-hospital complications and outcomes between positive and negative emotional events, our patient had a favorable course, with complete recovery after a year follow-up visit. (Figure 4)

## Discussion

TTS is frequently triggered by sudden and significant emotional stress, major physical illness, or trauma, which leads to the activation of the sympathetic nervous system. Catecholamines appear to play a central role in their pathophysiology. ^3^^-^^4^^)^ (Figure 1). Postmenopausal women constitute most TTS cases; male patients are not exempt but are often associated with physical triggers rather than emotional stress. The exact pathophysiology of TTS is unclear ^5^. Evidence suggests that happy heart syndrome (HHS) may require stronger stimuli to trigger a sufficiently high release of catecholamines compared to negative emotions, indicating a higher threshold for cardiovascular impact when happy events are processed. One hypothesized origin of TTS involves cognitive centers and the hypothalamic-pituitary-adrenal axis, which leads to significant catecholamine release, affecting the myocardium and coronary arteries. ^6^^,^^7^

**Figure 5:**
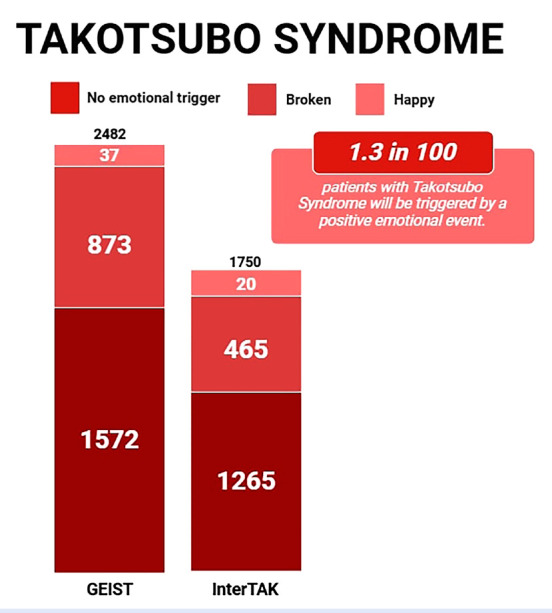
Prevalence of Takotsubo Syndrome as reported by the two largest registries. In the GEIST Registry (2482 patients), 1572 cases (63.3%) had no emotional trigger, 873 cases (35.1%) had a negative trigger (Broken Heart Syndrome), and 37 cases (1.4%) had a positive trigger (Happy Heart Syndrome). In the InterTAK Registry (1750 patients), 1265 cases (72.2%) had no emotional trigger, 465 cases (26.5%) had a negative trigger, and 20 cases (1.14%) had a positive trigger.

TTS is primarily triggered by physical stimuli, while 36% of TTS originates from emotional responses. Of these emotional triggers, 95.9% are due to negative events, while 4.1% are triggered by positive stimuli. ^7^ (Figure 5). Patients with emotional stress demonstrate significantly better in-hospital and long-term survival rates compared to those with physical triggers. ^(^^8^

Elevated cardiac biomarkers are commonly observed in TTS, as seen in our case, where levels were markedly elevated. CK-MB can be used in the initial workup; its diagnostic role is now limited. High-sensitivity Troponin is recommended for the diagnosis of TTS. However, NT-ProBNP is considered a more specific diagnostic biomarker than troponin. ^8^ In our case, both biomarkers were markedly elevated.

On ECG, the ST-segment elevation in the anterior precordial leads is the most common pattern in TTS. However, no well-defined ECG pattern exists for HHS cases. Notably, our patient showed ST-elevation in the inferior leads with Q-waves, which is uncommon in typical TTS. ^9^ Alterations in the inferior leads are more commonly observed in midventricular dysfunction cases, which typically present with ST-segment depression rather than elevation, a normal QTc interval, and no T-wave inversion. This pattern was consistent with our patient´s ECG findings. ^10^

Ventriculography patterns in TTS can vary based on the origin and location of the lesions. HHS is associated with a higher prevalence of atypical midventricular dysfunction than typical TTS. However, the most common pattern in both typical TTS and HHS is apical dysfunction, which was observed in our case. ^9^^,^^10^

In conclusion, prompt clinical assessment following intense emotional episodes, regardless of their nature, is crucial for timely intervention and enhanced patient care. Our atypical case of HHS highlights the diverse nature of TTS and broadens the spectrum of its triggers, including positive emotional events like a birthday present. Recognizing that various triggers can lead to TTS can improve screening and accurate diagnosis, advancing precision medicine. Identifying patient clusters with common features can help tailor management and expedite diagnosis. Further research into these unusual cases and their different presentations is essential for a better knowledge and understanding of this atypical form of TTS.

## References

[1] Singh T, Khan H, Gamble DT, Scally C, Newby DE, Dawson D (2022). Takotsubo Syndrome Pathophysiology, Emerging Concepts, and Clinical Implications. Circulation.

[2] Saposnik G, Baibergenova A, Dang J, Hachinski V (2006). Does a birthday predispose to vascular events. Neurology.

[3] Stiermaier T, Walliser A, El-Battrawy I, Pätz T, Mezger M, Rawish E (2022). Happy Heart Syndrome Frequency, Characteristics, and Outcome of Takotsubo Syndrome Triggered by Positive Life Events. JACC Heart Fail.

[4] Akashi YJ, Goldstein DS, Barbaro G, Ueyama T (2008). Takotsubo cardiomyopathy a new form of acute, reversible heart failure. Circulation.

[5] Lyon AR, Bossone E, Schneider B, Sechtem U, Citro R, Underwood SR (2016). Current state of knowledge on Takotsubo syndrome a Position Statement from the Taskforce on Takotsubo Syndrome of the Heart Failure Association of the European Society of Cardiology. Eur J Heart Fail.

[6] Lyon AR, Citro R, Schneider B, Morel O, Ghadri JR, Templin C, Omerovic E (2021). Pathophysiology of Takotsubo Syndrome JACC State-of-the-Art Review. J Am Coll Cardiol.

[7] Ghadri JR, Sarcon A, Diekmann J, Bataiosu DR, Cammann VL, Jurisic S (2016). InterTAK Co-investigators . Happy heart syndrome: role of positive emotional stress in takotsubo syndrome. Eur Heart J.

[8] Templin C, Ghadri JR, Diekmann J, Napp LC, Bataiosu DR, Jaguszewski M (2015). Clinical Features and Outcomes of Takotsubo (Stress) Cardiomyopathy. N Engl J Med.

[9] Ghadri JR, Wittstein IS, Prasad A, Sharkey S, Dote K, Akashi YJ (2018). International Expert Consensus Document on Takotsubo Syndrome (Part II) Diagnostic Workup, Outcome, and Management. Eur Heart J.

[10] Padilla-Lopez M, Duran-Cambra A, Belmar-Cliville D, Soriano-Amores M, Arakama-Goikoetxea S, Vila-Perales M (2023). Comparative electrocardiographic analysis of midventricular and typical takotsubo syndrome. Front Cardiovasc Med.

